# Factors Associated to Apical Root Resorption after Orthodontic Treatment

**DOI:** 10.2174/1874210601812010331

**Published:** 2018-04-30

**Authors:** João Dalto Viganó Pastro, Adriana Cândida Albuquerque Nogueira, Karina Maria Salvatore de Freitas, Fabricio Pinelli Valarelli, Rodrigo Hermont Cançado, Renata Cristina Gobbi de Oliveira, Ricardo Cesar Gobbi de Oliveira

**Affiliations:** Department of Orthodontics, Uningá University Center, Maringá, Brazil

**Keywords:** Root resorption, Bruxism, Orthodontics, Thumb sucking habit, Tongue thrusting habit, Onychophagia

## Abstract

**Objective:**

The aim of this study was to assess the possible factors associated to root resorption, common to daily clinical orthodontics, especially parafunctional habits.

**Methods:**

A retrospective study of 600 patients (308 females and 292 males) previously treated orthodontically was conducted. The sample was divided into two groups related to the degree of root resorption at the ending of treatment according to Malmgren. Group 1 comprised 507 patients with a mean initial age of 14.21 years and who had absent or mild final external root resorption, characterized by grades 0, 1 and 2 of root resorption; Group 2 comprised 93 patients with initial mean age of 14.57 years and who had moderate or severe root resorption, characterized by grade 3 and 4. The groups were then compared in terms of age at the beginning and ending of the treatment, treatment time, gender, type of treatment (with and without extractions), parafunctional habits (bruxism, onychophagia, the habit of biting objects, tongue thrusting habit and thumb sucking habit), allergies and pretreatment root resorption.

**Results:**

The results show that the initial age, gender, type of malocclusion, parafunctional habits and allergies do not represent a statistically significant risk of root resorption.

**Conclusion:**

Treatment time and type (with and without extractions) and the presence of external root resorption at the beginning of the treatment showed significant differences.

## 1 INTRODUCTION AND OBJECTIVE

Conceptually, Orthodontics is characterized by moving teeth inside the bone tissue. To promote this movement, a force is applied to the teeth and their periodontium taking them to the correct position and normal occlusion.

The orthodontic forces applied to the teeth cause biological stresses on the periodontal ligament, acting equally and simultaneously on both the alveolar bone and cementum. If these tissues, bone and cementum have a similar biological behavior, both must be equally resorbed during tooth movement [1, 2].

Apical root resorption is an undesirable side effect, otherwise frequent, of the orthodontic treatment [3-6]. It is defined as either a physiologic or pathological process resulting in the permanent loss of the cementum and dentine [7]. Its cause is related to local and/or mechanical factors, despite of other factors [8-10].

It was related to Orthodontics, firstly in 1914, by Ottolenghi [11] and since then, it has instigated the researchers to search uninterruptedly for the possible causes of this serious iatrogenic problem [12].

According to Consolaro [4], Apical Root Resorptions (ARR) are part of the biological cost of the orthodontic treatment; notwithstanding, they should not be considered as either normal or physiologic, but clinically acceptable. Some approaches and guidelines may prevent the occurrence of this pathology during orthodontic mechanics, thereby avoiding or reducing the number of teeth affected or its severity.

The diagnosis is generally performed by periapical radiographs [7, 4, 10, 13, 14] and they are normally asymptomatic; only when partial root loss due to severe resorption occurs is when the function and retention of the affected teeth may be compromised [15, 16].

Because of this constant concern of orthodontists regarding this clinical situation, the aim of this study was to compare the degree of root resorption after orthodontic treatment in patients with or without parafunctional habits, such as bruxism, onychophagia, the habit of biting objects, tongue thrusting and thumb sucking habit.

In addition to the habits, the following variables are related to apical root resorption: age at the beginning and ending of the orthodontic treatment, treatment time, gender, malocclusion type (according to Angle classification), treatment type (with or without tooth extractions), allergies and the presence of apical root resorption prior to orthodontic treatment.

## 2 MATERIALS AND METHODS

The study was approved by the ethics in Human Research Committee at UNINGÁ University Center, Maringá, PR, Brazil (CAAE 0184.0.362.000-09).

This study was conducted through the analysis of the files of the patients treated at a Graduation Orthodontic Clinic at UNINGÁ, Bauru, SP, Brazil. One thousand, forty hundred and thirty-seven files were analyzed, and 600 were selected for the sample. All patients selected were treated with fixed orthodontic appliances, by the same methodology, by the orthodontic graduate students of the dental school. The sample was divided into two groups: group 1 – absent to mild root resorption; group 2 – moderate to severe root resorption. The groups were compared to evaluate the factors predisposing to root resorptions which could be common to both groups. Inclusion criteria comprised: anamnesis, planning and clinical procedure sheets properly filled in; periapical radiographs of the maxillary and mandibular incisors, at the beginning and end of the orthodontic treatment, at excellent storage conditions.

The anamnesis sheet should contain the patient’s name, gender, birth date, malocclusion type according to angle, and the recording of the presence or absence of the parafunctional habits and allergies. The following habits were studied: bruxism, onychophagia, the habit of biting objects, tongue thrusting and thumb sucking habit. The periapical radiographs were evaluated by a single examiner at all moments, with the use of conventional negatoscope.

To assess the bruxism habit, this study was based on the patient and/or parent’s reports on the presence of clenching, grinding, muscle and/or Temporomandibular Joint (TMJ) pain at the morning. The evaluation of the tongue thrusting habit had also been performed during the anamnesis and clinical examination of the patients.

It identified the planning of tooth extractions during the treatment. All the treatments were identified by the number of teeth extracted, but for this study, we opted to divide them into treatments with and without extractions.

In the analysis of the periapical radiographs of the maxillary and mandibular incisors of the beginning and end of the orthodontic treatment, with the aid of a negatoscope, we ignored the teeth not showing normality parameters, for example, endodontic treatment and tooth reimplantation. For this present study, tooth exhibiting the highest degree of resorption of all teeth was considered.

The teeth were classified according to the scores proposed by Malmgren [17] to assess ARR severity because it is a visual qualitative method that despite of the fact of being relatively subjective, it has the advantage of not depending on the standardization of the radiograph [14].

**Fig. (1):**
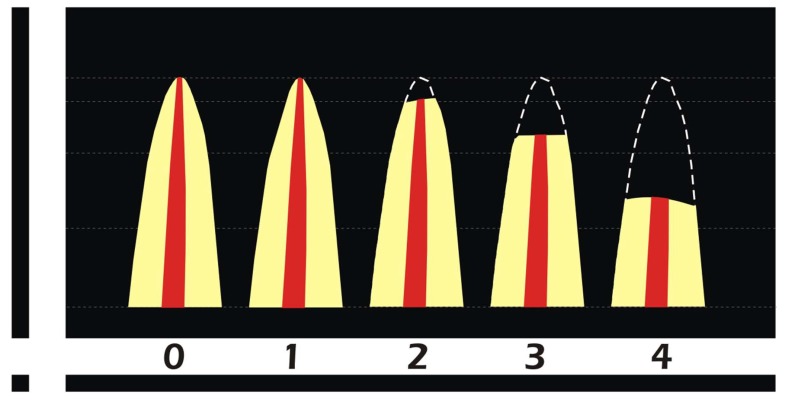
Root resorption classification score according to Malmgren [17]. Degree 0 - absence of resorption; Degree 1 - irregularity in the apical root contour; Degree 2 – resorption of up to 2 mm; Degree 3 – resorption from 2 mm up to 1/3 of the root; Degree 4 - loss greater than 1/3 of the root length.

The root resorption (Fig. **1**) scores as proposed by Malmgren [17] are:

- Degree 0 – the absence of resorption;
- Degree 1 – irregularity in the apical root contour, maintaining the original root length;
- Degree 2 – resorption of up to two millimeter of the root length;
- Degree 3 – resorption from 2 mm up to 1/3 of the root length; and
- Degree 4 – severe root resorptions, above 1/3 of the root length.

For every patient evaluated, 4 scores were assigned: 2 for initial ARR and 2 for final ARR of the maxillary and mandibular central incisors. Notwithstanding, this study considered the highest degree of ARR both for the initial and final records. After the identification of the resorption degree, the patients were divided into two groups according to the final ARR degree: groups 1 and 2. Group 1 was characterized by an absent to mild final ARR, with the patients showing degrees 0, 1 and 2 in the final ARR being selected. Group 2 was characterized by a moderate to severe final ARR, comprising patients exhibiting degrees 3 and 4 in final ARR. This division was performed because we considered that the degrees 1 and 2 of the final ARR did not display clinical significance for the patient after the treatment, once some ARR degree was almost expected at 100% of the cases orthodontically treated. On the other hand, the degrees 3 and 4 exhibited a high clinical significance, and they could be related to tooth mobility and even to tooth loss. Then, the groups were compared regarding the age at the beginning and end of the treatment, gender, treatment time, angle malocclusion type, treatment type (with or without extractions), parafunctional habits (bruxism, onychophagia, habit of biting objects, tongue thrusting habit and thumb sucking habit), allergies and ARR prior to the treatment, to verify the predisposition to ARR at the end of the orthodontic treatment.

### 2.1 Statistical Analysis

To verify the reliability of the results, the measurement was performed again on the periapical radiographs of the maxillary and mandibular incisors at the beginning and end of the treatment of 80 patients randomly selected after a time interval greater than 60 days. The scores obtained at the first and second evaluations were submitted to weight Kappa test.

#### 2.1.1 Statistical Analysis between Groups and Variables

For intergroup comparisons of the final ARR regarding the gender, malocclusion type, treatment type, bruxism, biting objects, tongue thrusting, thumb sucking, allergies, and the presence of initial resorption, Chi-square test was applied. To correlate ARR with treatment time and initial age, independent t test was applied. To estimate ARR probability in the patients treated with extraction in relation to those treated without extraction, a logistic regression was performed.

## 3 RESULTS

Group 2 (severe resorption) presented both the final age and the treatment time significantly longer than Group 1 (Table **1**).

Concerning treatment type and ARR prior to the orthodontic treatment, the final root resorption was significant. There were no significant differences regarding the other variables: gender, malocclusion type, bruxism, onychophagia, biting objects, tongue thrusting and thumb sucking (Table **2**).

Table **3** exhibits the logistic regression results, performed to obtain the probability ratio of ARR among the patients treated with and without tooth extraction between Groups 1 and 2. Patients treated with tooth extraction had a probability of 2.72 times greater on undergoing ARR (Odds ratio = 2.71875)

## 4 DISCUSSION

It is known that the ideal research would be a prospective study. However, this study is valid because, as far as we are concerned, little information is available on the literature about this issue. Additionally, this study evaluated a large number of patients.

Also, the study was conducted at a Dental School with a large number of patients treated, displaying complete files properly filled in by the graduate orthodontic students. Therefore, the study was based on the information provided by the patients or their parents within their files.

It is important to emphasize the difficulty in finding a sample as large as that of this present study meeting the inclusion criteria assigned for it: anamnesis, planning, and clinical procedure sheets fully recorded; periapical radiographs of the maxillary and mandibular incisors at the beginning and end of the treatment.

Accordingly, we verified about 1,437 files, of which 600 met the inclusion criteria, comprising 292 males and 308 females, with almost 50% of the total sample for each gender, which favored the sample quality. The clinical examinations to detect parafunctional habits had also been executed by the post-graduate students at the patient’s first appointment together with the filling of the anamnesis sheet. Notwithstanding, concerning the bruxism evaluation, some failure could have occurred because this information was reported by the patients or their parents and it may not be perceived by either them or the orthodontists because of lack of signs and symptoms. However, since at the moment of the study these data were accessible and because of the lack of information on the literature regarding this subject, the results of this present study can be considered. Moreover, bruxism was not clinically evaluated because of the failure in considering the tooth weariness assessment as a determining factor in its diagnosis; because it is known that some patients do not exhibit them and have parafunctional habit. Also, other factors may be related to tooth weariness.

There was not a correlation between the initial age and resorption (Table **2**). However, this result should be cautiously interpreted because the final mean ages between groups were very close to each other, as already observed by other studies [16] Youth and adults are at the same risks for ARR, according to a study conducted in 2005 [4]. Notwithstanding, adults who had already undergone chronic inflammatory periodontal disease showed sequelae, such as smaller alveolar bone ridge height and higher clinical crown, therefore modifying the crown/root ratio. These are the features which would increase the ARR risks, instead of the age as an isolated factor.

Patients presenting severe resorptions exhibited a higher average treatment time than patients with slight ARR (Table **1**), suggesting that longer treatment time periods would be at higher risk of severe ARR than shorter treatment time periods. This result corroborates the findings of several authors who pointed out that the treatment time directly influences the development of root resorption [8, 13, 18, 19]. It happens because the longer the treatment time, the greater will be the teeth movement and possibly greater root resorption will occur. However, treatment time would not be the main factor for ARR risk. Several authors affirmed based on scientific evidences that the treatment time itself would not have a positive correlation with root resorption [4, 18, 19]. ARR would be correlated with a greater amount of movements which may occur in longer treatments and not only with treatment length [18, 20].

There was no relationship between gender and severe ARR (Table **1**). Both genders may or may not be equally affected by severe ARR at the end of the treatment. This information is in agreement with that of the literature [4, 13, 21].

There was no relationship between the malocclusion type and severe ARR (Table **1**). Other authors have already discharged the possible relationship of severe ARR with the malocclusion type [8, 19, 21]. ARR would be correlated instead with the malocclusion severity, because they require greater amount of movement and therefore would be more susceptible to severe ARR occurrence [2, 20, 22].

Cases with severe ARR are treated more frequently with tooth extraction (Table **2**). Accordingly, in these cases, a higher amount of tooth movement is required, consequently increasing the duration of force application and therefore provoking higher resorption. Patients submitted to tooth extractions have 2.72 times more chances of developing severe ARR, according to the logistic regression test specifically applied for this study variable, between a confidence interval of 1.72 and 4.29 (Table **3**).

Orthodontic therapy through extractions is the main sequelae with frequent presence of resorptions at the end of treatment [2, 23, 24]. Several authors corroborate this and suggest that extraction treatments are more prone to severe ARR because of the retraction mechanics of the anterior teeth causing greater movement of the root apexes and requiring longer treatment time [25, 26].

There was no relationship between bruxism and severe ARR (Table **2**). As far as we are concerned, little studies related bruxism with ARR. In 2004, a study affirmed that bruxers with clinical signs of tooth weariness showed teeth with higher rates of root resorptions than non-bruxers The authors considered degree 2 of Malmgren score to identify ARR and they did not find any tooth with degrees 3 and 4 [27].

Nail biting habit showed a relatively high incidence, of 33.17% of the patients of the sample, but it was not statistically significant for the development of severe ARR (Table **2**). Onychophagia could be capable of increasing root resorption during the orthodontic treatment, when its intensity and frequency are considered [2]. Despite the controversial role of this habit in ARR, cases in which the resorption process is maintained after the appliance removal, the presence of onychophagia should be checked to eliminate a possible interference [4].

The habit of biting objects was not statistically significant for ARR development (Table **2**). Although this habit produces as much as the same intrusive forces on the teeth, object biting difficulty becomes a chronic habit such as tongue thrusting and onychophagy, for example. Probably, this is the reason why it does not exhibit higher ARR risks.

Atypical swallowing, characterized by tongue thrusting was not statistically significant for the increase of severe ARR incidence (Table **2**). However, among all variables not presenting statistical significance for ARR, this was the one closer to reach it (*P*=0.06). Probably, this occurred because of the intrusive and constant movement produced by this habit on the teeth.

Tongue thrusting habit associated with anterior open bite was reported as a factor which could increase ARR risks [21]. Some authors, in 1992, by analyzing root resorptions of maxillary central incisors of patients presenting open bite or marked overbite, found a smaller root length in the patients with open bite even prior to the orthodontic treatment [28]. After the orthodontic treatment, the patients with the open bite still maintained ARR degrees significantly higher than those presenting marked overbite. These authors concluded that oral forces, such as tongue thrusting, accounted for injury of the root integrity. The orthopedic force exerted by the tongue can be compared to that of either intrusive or torque mechanics. For this reason, patients with anterior open bite can have their root length decreased, even prior to orthodontic treatment. The persistent trauma, such as tongue thrusting during the orthodontic treatment may result in an increase of ARR at the end of treatment [29].

Thumb sucking was also not statistically significant for the development of severe ARR (Table **2**), with little incidence in this study sample, probably because of the high initial age mean of the groups, between 14.21 and 14.57 years, where due to social reasons, this habit is not frequent. It has been suggested that longer thumb sucking, similarly to tongue thrusting, may influence either directly (by increasing the overjet) or indirectly (through tooth movements) on ARR [29].

The allergies were not statistically significant for severe ARR (Table **2**). None study methodologically well planned and executed could find any influence of both systemic alterations and medicament use on root resorption [4]. The cementoblasts do not have receptors for systemic mediators and are not targets of the secondary action of these medicaments; this way, root resorption causes are strictly local [4].

Patients exhibiting some ARR degree prior to orthodontic treatment, showed significant rates of severe ARR (Table **2**). Patients with any ARR degree (on initial periapical radiographs) are more prone to develop severe ARR. Therefore, the clinicians should be aware of this fact and the treatment should only be carried out after careful analysis of the periapical radiographs prior to appliance installation. Other authors have already affirmed that teeth exhibiting previous signs of root resorption are more prone to severe ARR [2, 26, 30].

Severe ARR should be a concern for the orthodontist. Cautious should be taken in an attempt to prevent or control the occurrence of root resorption and special attention should be given in treatments with extractions, or in cases presenting previous ARR degree.

During treatment planning, techniques resulting in faster treatments, obviously within biological limits of force application, should be chosen in an attempt of reducing the force amount and the occurrence of severe ARR. Extractions should be indicated only when strictly necessary [4].

Radiographs are mandatory to control and prevent root resorptions. At the beginning of the treatment, a periapical evaluation of all teeth should be executed; it is recommended to repeat the examination every six months, at least for the maxillary and mandibular incisors [31]. No tooth movement is resorption-free, even the simpler ones [13, 20]. The orthodontist should be aware of any situation in which root resorption risks are imminent.

## CONCLUSION

Based on the methodology used and on the results found, it can be concluded that:

- The age at the beginning of the treatment, gender, malocclusion type, parafunctional habits and allergies are not risk factors for root resorption.
- It is considered as risk factor for root resorption, the cases treated with tooth extractions, root resorption prior to the beginning of the treatment and treatment time.

## ETHICS APPROVAL AND CONSENT TO PARTICIPATE

The study was approved by the ethics in Human Research Committee at UNINGÁ University Center, Maringá, PR, Brazil (CAAE 0184.0.362.000-09).

## HUMAN AND ANIMAL RIGHTS

No animals were used in this research. All research procedures followed were in accordance with the ethical standards of the committee responsible for human experimentation (institutional and national), and with the Helsinki Declaration of 1975, as revised in 2008.

## Figures and Tables

**Table 1:** Intergroup comparison of the ages at the initial and final stages of orthodontic treatment and treatment time (independent t test).

**Variables**	**GROUP 1** **Mild Resorption** **N = 507**		**GROUP 2** **Moderate to Severe Resorption** **N = 93**		***P***
	**MEAN**	**SD**	**MEAN**	**SD**	
**Initial age (years)**	14.21	2.45	14.57	2.67	0.203
**Final age** **(years)**	16.02	2.56	16.99	2.97	0.001*
**Treatment Time (years)**	1.81	0.83	2.41	0.99	0.000*

* Statistically significant for *P* < 0.05.

**Table 2:** Intergroup comparison of gender, malocclusion type, treatment type, bruxism, onychophagia, object biting, tongue thrusting, thumb sucking, allergies and presence of initial resorption (Chi-square).

**Variables**		**GROUP 1** **Mild Resorption** **N = 507**	**GROUP 2** **Moderate to Severe Resorption** **N = 93**	**λ**	**DF**	***P***
**GENDER**	MALE	252	40	1.40	1	0.235
	FEMALE	255	53			
**MALOCCLUSION**	CLASS l	220	38	0.26	2	0.877
	CLASS ll	269	52			
	CLASS lll	18	3			
**TYPE OF TREATMENT**	WITH EXTRACTION	192	58	19.40	1	0.000*
	WITHOUT EXTRACTION	315	35			
**BRUXISM**	YES	12	2	0.01	1	0.898
	NO	495	91			
**ONYCHOPHAGY**	YES	163	36	1.52	1	0.216
	NO	344	57			
**BITING HABITS**	YES	39	12	2.74	1	0.097
	NO	468	81			
**TONGUE THRUSTING**	YES	53	16	3.51	1	0.060
	NO	454	77			
**SUCKING**	YES	25	4	0.06	1	0.794
	NO	482	89			
**ALLERGIES**	YES	213	46	1.77	1	0.182
	NO	294	47			
**ARR at T1**	0	181	34	42.16	3	0.000*
	1	322	48			
	2	4	8			
	3	0	3			
	4	0	0			

*Statistically significant for *P*<0.05

**Table 3:** Logistic regression for the probability estimative of patients undergoing extraction to be at higher risk of ARR in the comparison between Group 1 and 2.

**N=600**	**Model: logistic regression (logit) N of 0’s: 93 (DADOS DISSERTACAO Dep. var: Grupo T1 Loss: Max likelihood (MS-err. Scaled to 1)** **Final loss: 249. 19946411 Chi^2^(1)=19.143 *p*=.00001**	
	**Const. B0**	**Exodontia**
Estimate	-2.197225	1.000172
Standard Error	0.1785125	0.2327512
t(598)	-12.30852	4.297174
*p*-level	0	0.0000201912
-95%CL	-2.547812	0.5430631
+95%CL	-1.846637	1.457281
Wald’s Chi-square	151.4997	18.4657
*p*-level	0	0.00001735002
Odds ratio (unit ch)	0.1111111	2.71875
-95%CL	0.07825268	1.721271
+95%CL	0.1577668	4.294269
Odds ratio (range)	-	2.71875
-95%CL	-	1.721271
+95%CL	-	4.294269
